# Development of Alumina–Mesoporous Organosilica Hybrid Materials for Carbon Dioxide Adsorption at 25 °C

**DOI:** 10.3390/ma11112301

**Published:** 2018-11-16

**Authors:** Chamila Gunathilake, Rohan S. Dassanayake, Chandrakantha S. Kalpage, Mietek Jaroniec

**Affiliations:** 1 Department of Chemical and Processing Engineering, Faculty of Engineering, University of Peradeniya, Peradeniya 20400, Sri Lanka; chamilag@pdn.ac.lk (C.G.); csk@eng.pdn.ac.lk (C.S.K.); 2 Department of Chemistry and Biochemistry, Kent State University, Kent, OH 44242, USA; 3 Department of Chemistry, Ithaca College, Ithaca, NY 14850, USA; rdassanayake@ithaca.edu

**Keywords:** alumina, CO_2_ capture, porous hybrid adsorbents, mesoporous organosilica

## Abstract

Two series of alumina (Al_2_O_3_)–mesoporous organosilica (Al–MO) hybrid materials were synthesized using the co-condensation method in the presence of Pluronic 123 triblock copolymer. The first series of Al–MO samples was prepared using aluminum nitrate nanahydrate (Al–NN) and aluminum isopropoxide (Al–IP) as alumina precursors, and organosilanes with three different bridging groups, namely tris[3-(trimethoxysilyl)propyl]isocyanurate, 1,4-bis(triethoxysilyl)benzene, and bis(triethoxysilyl)ethane. The second series was obtained using the aforementioned precursors in the presence of an amine-containing 3-aminopropyltriethoxysilane to introduce, also, hanging groups. The Al–IP-derived mesostructures in the first series showed the well-developed porosity and high specific surface area, as compared to the corresponding mesostructures prepared in the second series with 3-aminopropyltriethoxysilane. The materials obtained from Al–NN alumina precursor possessed enlarged mesopores in the range of 3–17 nm, whereas the materials synthesized from Al–IP alumina precursor displayed relatively low pore widths in the range of 5–7 nm. The Al–IP-derived materials showed high CO_2_ uptakes, due to the enhanced surface area and microporosity in comparison to those observed for the samples of the second series with AP hanging groups. The Al–NN- and Al–IP-derived samples exhibited the CO_2_ uptakes in the range of 0.73–1.72 and 1.66–2.64 mmol/g at 1 atm pressure whereas, at the same pressure, the Al–NN and Al–IP-derived samples with 3-aminopropyl hanging groups showed the CO_2_ uptakes in the range of 0.72–1.51 and 1.70–2.33 mmol/g, respectively. These data illustrate that Al–MO hybrid materials are potential adsorbents for large-scale CO_2_ capture at 25 °C.

## 1. Introduction

Among many heat-trapping gases, including CO_2_, SO_2_, NO, N_2_O, NO_2_, and CH_4_, carbon dioxide (CO_2_) is considered one of the major contributors to global warming. According to the report released by EPA in 2011, the contribution of CO_2_ is about 84% of the total discharge of gases into the atmosphere. Currently, the CO_2_ concentration exceeded 400 ppm, and the average global temperature has already increased by 1–2 °C, as compared to the values reported over the last few decades. Although this temperature fluctuation seems to be small, it has a significant effect on the global climate patterns, leading to the melting of glaciers in north and south poles, eventually causing the sea level to rise. Thus, reducing the concentration level of CO_2_ is an urgent requirement to keep the atmosphere safe and diminish the effects of global warming. 

Nowadays, the preferred technology for industrial CO_2_ capture involves chemical absorption in aqueous solutions of amines. Alkanolamines, including monoethanolamine (MEA) and diethanolamine (DEA), are commonly used in this process [1]. However, the amine-based scrubbing techniques possess several drawbacks. For instance, the regeneration of absorbed CO_2_ from liquid amine is energy intensive, and replacement of amine after several cycles is needed due to its degradation under oxidizing and reducing chemical environments. 

Solid sorbents have attracted a great attention as potential alternatives to the liquid amine scrubbing techniques, due to their high stability, tunability, and reusability. Unlike aqueous liquid amines, porous solid sorbents adsorb CO_2_ via physisorption (physical adsorption) [2] or chemisorption (chemical adsorption) [2], and avoid many drawbacks associated with liquid amines [3,4]. Chemisorption on solid sorbents is achieved by modifying their surface with basic sites such as alkaline carbonates and different amine functional groups, which show high affinity toward acidic CO_2_. Physical solid sorbents for CO_2_ capture include activated carbons [5,6], carbon molecular sieves [7,8], carbon nanotube-based sorbents [9,10], zeolites [11,12], and metal-organic frameworks [13,14]. Materials like alkali metal-based sorbents [15,16,17,18], amine-functionalized solid sorbents such as activated carbons [19,20], carbon nanotubes [21,22], solid resins [23], modified zeolite-based sorbents [24,25], polymer-based sorbents [26,27], amine-functionalized silica [28,29], grafted silica sorbents [30,31,32], and amine-impregnated alumina sorbents [33], have been investigated as chemical solid sorbents.

Mesoporous silica materials, such as SBA-15 [34] and MCM-41 [35], have been investigated for CO_2_ capture. However, these materials show low CO_2_ sorption capacities due to weak interactions between silanol groups and CO_2_. Therefore, MCM-41 and SBA-15 silica materials modified with different amine groups, including diethylenetriamine, tetraethylenepentamine, polyethyleneimine, and ethylenediamine, have been studied for CO_2_ capture [30,31,32,36,37,38,39,40]. The chemical incorporation of amine groups onto silica matrix can be achieved either by co-condensation [41,42] or post-synthesis grafting [31,43,44]. Typically, the higher loading of amine groups leads to higher CO_2_ capacity depending on the properties of mesoporous silica. Thus, the selection of proper silica support with high porosity and high surface area before amine grafting is essential. During grafting of amine groups onto silica surface, the surface silanols react with aminosilanes. Therefore, maintaining high surface silanol density is beneficial for achieving higher loading of amine groups and larger CO_2_ sorption capacity. 

Over the past few years, the amine-grafted mesoporous silica and impregnated alumina materials have been investigated as solid sorbents for CO_2_ capture [45,46]. Mesoporous silica inherits high surface area, tunable, and well-defined porosity, which are important for uniform grafting of amine functional groups. Similarly, alumina materials show favorable properties, including high surface area, high porosity, and thermal and mechanical stability, which are suitable for amine modification. The adsorption properties of alumina strongly depend on its structure and thermal treatment temperature (calcination temperature). For example, mesoporous alumina materials feature large pore volume, three-dimensional (3D) interconnected mesoporosity, and thermal stability. Due to these unique characteristics, alumina-based materials have been investigated for gas adsorption applications. Examples of alumina-based materials for CO_2_ capture include amine-impregnated Al_2_O_3_ [33], basic Al_2_O_3_ [46], amine-modified mesoporous Al_2_O_3_ [47,48], MgO/Al_2_O_3_ [49], and Al_2_O_3_–organosilica [50] materials. 

Interestingly, the adsorption properties of alumina–mesoporous silica composites can be further improved by introducing silica precursors with nitrogen-containing functional groups, such as amines and organic bridging groups [50,51]. We previously reported the synthesis of alumina–mesoporous organosilica hybrid materials with isocyanurate bridging groups and their CO_2_ adsorption properties at elevated temperatures [50]. Incorporation of organic bridging groups facilitated the formation of crosslinked mesostructures with well-ordered pores and enhanced surface properties [51]. In addition, the organic moieties of bridging groups increase hydrophobicity of the mesoporous silica matrix, which is beneficial for CO_2_ sorption. 

**Scheme 1:**
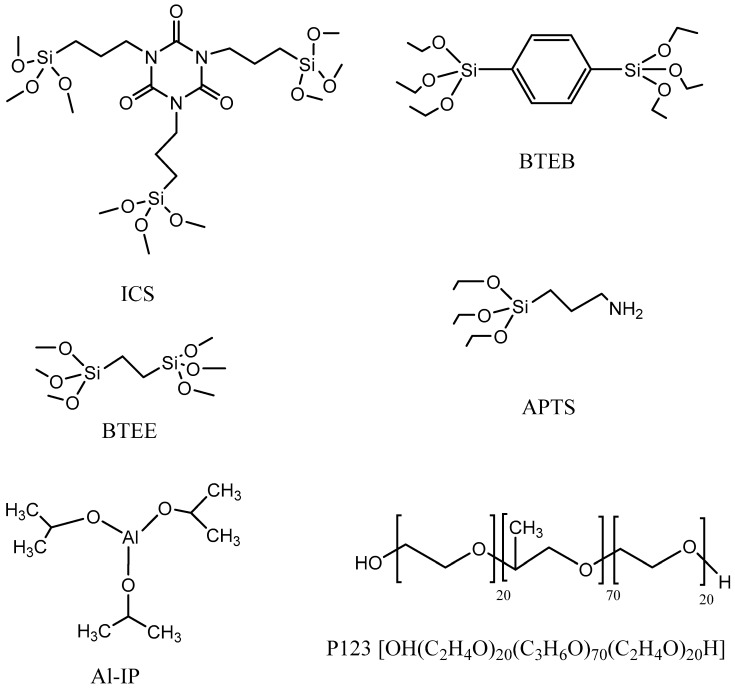
Structures of the chemicals used in the synthesis of different Al–MO hybrid materials.

In this study, we report the synthesis and CO_2_ adsorption properties of mesoporous Al–MO hybrid materials at 25 °C. Here, we also present the effects of different alumina precursors, silica precursors with varying organic bridging groups, amine-based silica hanging groups, and calcination process on the CO_2_ capture at 25 °C. Al–MO hybrid materials were prepared in the presence of triblock copolymer Pluronic P123 using alumina and organosilica precursors. Two alumina precursors used in this study include aluminum nitrate nanahydrate (Al–NN) and aluminum isopropoxide (Al–IP), see Scheme 1. Silanes with three different bridging groups, including nitrogen-containing tris[3-(trimethoxysilyl)propyl]isocyanurate (ICS), and non-nitrogen-containing 1,4-bis(triethoxysilyl)benzene (BTEB), and bis(triethoxysilyl)ethane (BTEE)), were used as organosilica precursors. The grafting of amine groups was achieved by using 3-aminopropyltriethoxysilane (APTS), see Scheme 1. To our knowledge, this is the first report on the Al–MO hybrid materials prepared by using different alumina and silica precursors with various organic bridging groups and amine hanging groups for CO_2_ capture at ambient conditions. 

## 2. Materials and Methods 

### 2.1. Materials

Tris[3-(trimethoxysilyl)propyl]isocyanurate (ICS), 1,4-bis(triethoxysilyl)benzene (BTEB), bis(triethoxysilyl)ethane (BTEE), and 3-aminopropyltriethoxysilane (APTS) were purchased from Gelest Inc (Morrisville, PA, USA). Pluronic P123 (EO_20_PO_70_EO_20_) triblock copolymer was purchased from BASF Corporation (Florham Park, NJ, USA). Absolute ethanol was obtained from AAPER Alcohol and Chemical Company (Shelbyville, KY, USA). Ethanol (95%) was purchased from Fisher Scientific (Pittsburgh, PA, USA). Aluminum isopropoxide (Al–IP) and aluminum nitrate nanahydrate (Al–NN) were purchased from Sigma Aldrich (St. Louis, MO, USA). All reagents were in analytical grade, and used without further purification.

### 2.2. Synthesis Method

**Scheme 2:**
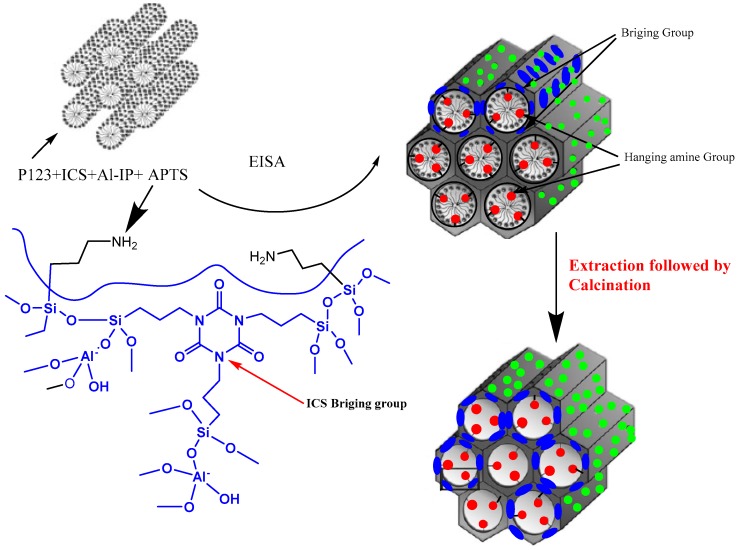
Illustration of the synthesis route for alumina–organosilica material (Al-IC10-AP10) using isocyanurate bridging silane (ICS) and 3-aminopropyltriethoxysilane (APTS).

Alumina–mesoporous organosilica (Al–MO) hybrid samples were synthesized using a slightly modified literature method [50,52]. The procedure used for the preparation of the first series of Al–MO hybrid materials was as follows: 1.0 g of Pluronic P123 (EO_20_PO_70_EO_20_) and known amounts of Al–NN (aluminum nitrate nanahydrate) or Al–IP (aluminum isopropoxide) were added to 20 mL of absolute ethanol in a 250 mL polypropylene bottle. Then, the reaction mixture was stirred at 25 °C for 70 min. After that, a predetermined amount of silica precursor with different organic bridging groups (ICS, BTEE or BTEB) was added to the resultant mixture. The same steps were used in the preparation of the second series of Al–MO hybrid materials with APTS hanging groups. A known amount of APTS was added to the reaction mixture after adding the silica precursor (ICS, BTEE, or BTEB). In both cases, the final mixture was stirred at room temperature for 4 h and kept in an oven at 60 °C for 48 h. Then, extraction of the P123 block copolymer template from the resulting white/yellow solid was performed using 55 mL of 95 % ethanol solution in a 250 mL polypropylene bottle under vigorous stirring for a few minutes. The resulting dispersion was kept in the oven (Sheldon Manufacturing Inc., Cornelius, OR, USA) at 100 °C for another 24 h. Next, the product was filtered and rinsed thoroughly with 95 % ethanol and dried in an oven at 70 °C for an additional 20 h. Finally, calcination of the solid obtained after extraction was conducted in a horizontal quartz tube furnace (Lindberg Blue M manufactured by Thermal Product Solutions, White Deer, PA, USA) at 350 °C for 2 h in flowing N_2_ at a heating rate of 2 °C/min. The samples obtained prior to the extraction step (as-synthesized), after extraction (extracted), and after extraction followed by calcination (extracted and calcined), were tested for their adsorption properties. Scheme 2 shows the route used for the synthesis of alumina–organosilica (Al-IC10-AP10) material using two organosilanes, the first with an isocyanurate bridging group (ICS) and the second with 3-aminopropyl (APTS) hanging group.

The aluminum content (0.011 mol) was kept constant for all samples studied. The number of moles of Si used in the synthesis was a percentage of the total number of Al moles. Based on our previous study, the optimum Si% for tris[3-(trimethoxysilyl)propyl]isocyanurate (ICS) and 3-aminopropyltriethoxysilane (APTS) was found to be 10% [50]. The Al–MO materials with higher Si% than 10 showed smaller pore widths due to the geometrical constraints created by bulky silane bridging groups during self-assembly process [50]. Therefore, Si percentage, with respect to the total number of Al moles, was maintained at 10% for all Al–MO samples. The samples were denoted based on the precursors used in the initial reaction mixture. For instance, the short abbreviation AN refers to aluminum nitrate nanahydrate (Al–NN), and AI to aluminum isopropoxide (Al–IP). Short abbreviations IC, B, E, and AP, were used for tris[3-(trimethoxysilyl)propyl]isocyanurate (ICS), 1,4-bis(triethoxysilyl)benzene (BTEB), bis(triethoxysilyl)ethane (BTEE), and 3-aminopropyltriethoxysilane (APTS), respectively. For instance, AN-B10 refers to the samples synthesized using Al–NN and BTEB (10% Si), whereas AN-B10-AP10 refers to the samples synthesized using Al–NN, and BTEB (10% Si) and APTS (10% Si). In both cases, Al–NN acts as an alumina precursor. Similarly, AI-B10 refers to the samples prepared using Al–IP and BTEB (10% Si), while AI-B10-AP10 refers to the samples obtained by using Al–IP, BTEB (10% Si), and APTS (10% Si)). The as-synthesized samples are denoted with a # mark. Labels for all extracted samples do not have an asterisk (*), whereas the samples subjected to the extraction, followed by calcination, are marked with an asterisk (*). 

Four analogous samples to AN-IC10, AN-IC10*, AN-IC10-AP10, and AN-IC10-AP10* were studied in a previous work [50], where a series of materials was prepared by varying the amount of ICS. The synthesis and calcination conditions used in [50] and this work are slightly different, which results in different adsorption parameters. For instance, AN-IC10 in the current paper was calcined at 350 °C with heating rate of 2 °C/min, while an analogous sample reported in [50] was calcined at 300 °C with heating rate of 3 °C/min. In previous work [50], alumina precursors were mixed with one bridging silane only, isocyanurate (IC), by varying its percentage from 10 to 80%, while, in this work, silanes (10% only) with different bridging were explored. Importantly, the previous work [50] was focused on the CO_2_ capture at elevated temperatures, while the current work reports the newly synthesized samples with different functional groups for CO_2_ adsorption at 25 °C. 

### 2.3. Characterization of Materials

Nitrogen (N_2_) adsorption isotherms were collected at −196 °C (77 K) using an ASAP 2010 volumetric analyzer (Micromeritics, Inc., Norcross, GA, USA). All materials were outgassed under vacuum at 110 °C for 2 h before recording the adsorption measurements. 

High-resolution thermogravimetric measurements were conducted using a TGA Q-500 analyzer (TA Instruments, Inc., New Castle, DE, USA). Thermogravimetric (TG) studies were performed from 25 °C to 700 °C in flowing N_2_ gas, using a heating rate of 10 °C/min. The TG profiles were used to study the thermal stability of the hybrid materials and the removal of P123 template.

### 2.4. NMR Analysis

All NMR spectra were collected on Bruker Avance (III) 400WB NMR spectrometer (Bruker Biospin Corporation, Billerica, MA, USA) equipped with a magic-angle spinning (MAS) triple resonance probe head having zirconia rotors of 4 mm diameter by using the procedure reported in [50].

### 2.5. CO_2_ Adsorption Measurements

CO_2_ adsorption studies were conducted using an ASAP 2020 volumetric adsorption analyzer (Micromeritics, Inc., Norcross, GA, USA) at the pressures up to ~1.2 atm and 25 °C using ultrahigh pure CO_2_ gas. All samples were outgassed at 110 °C for 2 h under vacuum before the analysis. The sample reusability was tested over a number of adsorption/desorption cycles for the sample with the highest CO_2_ adsorption capacity. Six consecutive adsorption/desorption runs were recorded on ASAP 2020. For instance, the first adsorption isotherm measurement was carried out up to 1.2 atm followed by desorption cycle. After the completion of the first run, the second adsorption/desorption run was started manually on the same instrument, and so on.

### 2.6. Calculation of Surface Properties

The Brunauer–Emmett–Teller (BET) specific surface area (S_BET_) of each sample was calculated by following its N_2_ adsorption isotherm in the relative pressure range of 0.05 to 0.20. The single-point pore volume (V_sp_) was determined based on the amount N_2_ gas adsorbed at a relative pressure of ~0.98. The pore size distributions (PSD) were calculated using adsorption branches of N_2_ adsorption/desorption isotherms and applying the improved Kruk–Jaroniec–Sayari (KJS) method developed for cylindrical pores [53]. The total pore volume (V_t_) was calculated by integrating the entire PSD curve and the volume of micropores (V_mi_) was calculated by taking the difference between V_t_ and V_meso_ (V_t_ − V_meso_). The mesopore volume (V_meso_) was determined by integrating the PSD curve from 2 nm to the end of the PSD curve. In addition to the PSD curves that display the overall distribution of pores, the pore widths (W_max_) corresponding to the maxima of these distributions are also tabulated to show the characteristic pore sizes of the samples studied. 

## 3. Results and Discussion

### 3.1. Properties of Al–MO Hybrid Composites

**Figure 1:**
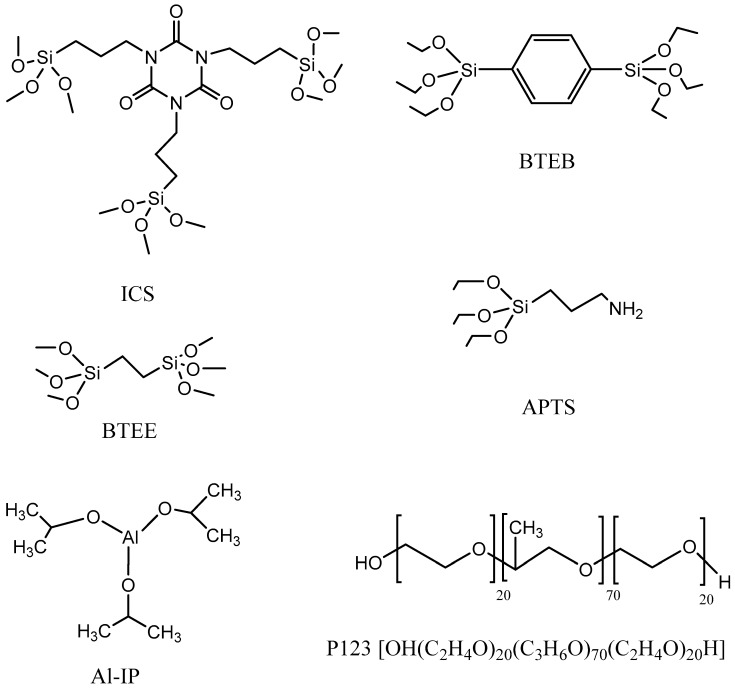
DTG profiles of the as-synthesized (^#^) alumina–silica composite with isocyanurate bridging groups and alumina–silica composites with isocyanurate bridging groups in the presence and absence of aminopropyl groups after extraction followed by calcination (*). Note: AN and AP refer to the aluminum nitrate and aminopropyl, respectively.

High-resolution thermogravimetry (HR-TG) and differential thermogravimetry (DTG) studies were conducted to identify the thermal stability of silica precursors with ethylene, benzene, isocyanurate bridging groups, and amine hanging groups. Figure 1 shows the DTG profiles recorded in flowing N_2_ for AN-IC10^#^, AN-IC10*, and AN-IC10-AP10* samples. As shown in Figure 1, all samples exhibit a sharp peak approximately at 100 °C, assigned to the loss of physically adsorbed water. The as-synthesized (AN-IC10^#^) sample displays two additional peaks on its DTG profile in the temperature ranges of 150–300 °C and 350–480 °C, corresponding to the decomposition of P123 block copolymer template and isocyanurate bridging groups, respectively, see Figure 1. 

**Figure 2:**
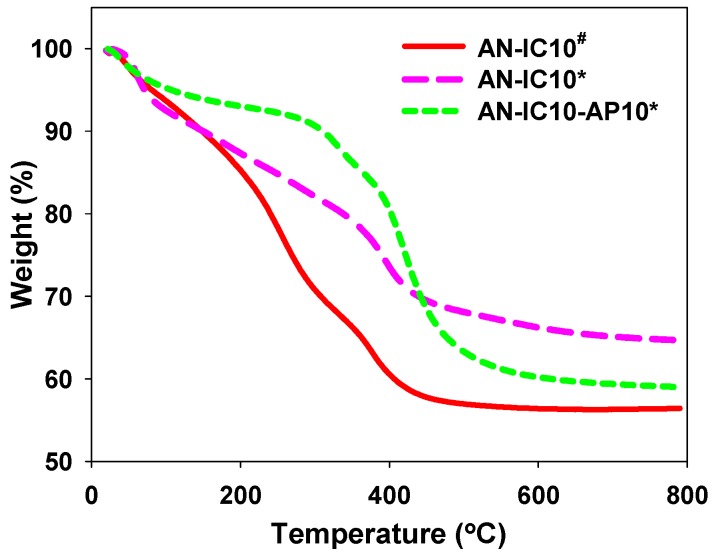
TG profiles of the as-synthesized (^#^) alumina–silica sample with isocyanurate bridging groups and alumina–silica samples with isocyanurate bridging groups in the presence and absence of aminopropyl groups after the extraction and calcination (*). Note that AN and AP refer to the aluminum nitrate and aminopropyl, respectively.

**Figure 3:**
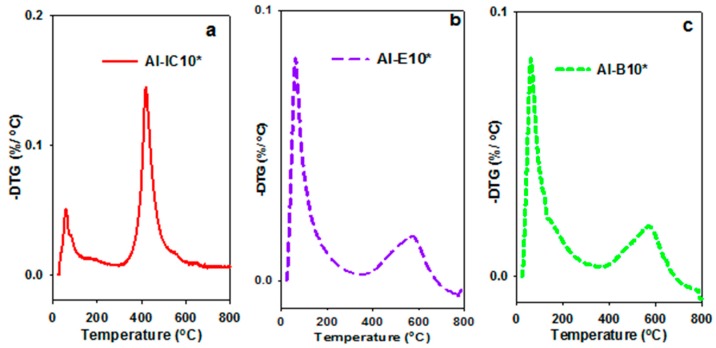
DTG profiles for the extracted and calcined (*) alumina–silica composites containing isocyanurate (IC; panel **a**), ethylene (E; panel **b**), and benzene (B; panel **c**) bridging groups. Note that AI refers to aluminum isopropoxide.

The DTG profiles obtained for the extracted and calcined alumina–silica samples (AN-IC10-AP10* and AN-IC10*) with isocyanurate (ICS) bridging groups in the presence and absence of aminopropyl group show a second major peak centered at 430 °C, related to the decomposition of isocyanurate bridging groups, see Figure 1. The small peak observed at 330 °C for AN-IC10-AP10* corresponds to the degradation of aminopropyl groups. The DTG profiles for AN-IC10-AP10* and AN-IC10* do not have any peak in the temperature range from 150 to 300 °C, indicating the complete removal of block copolymer template via extraction followed by calcination, see Figure 1. The corresponding TG curves of AN-IC10^#^, AN-IC10*, and AN-IC10-AP10* samples are shown in Figure 2. Figure 3 displays the DTG profiles of three extracted and calcined alumina–silica samples (AN-IC10*, AN-E10*, and AN-B10*) with ICS, BTEE, and BTEB bridging groups. As can be seen from Figure 3, peaks at ~400, 550, and 600 °C correspond to the thermal degradation of isocyanurate, benzene, and ethylene bridging groups, respectively. 

**Figure 4:**
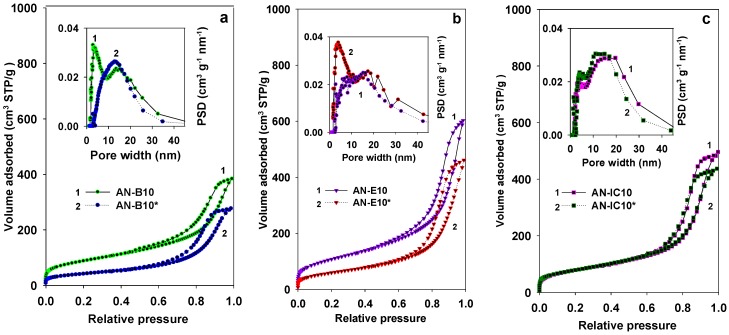
N_2_ adsorption isotherms and their corresponding PSD curves (insets) for the extracted (AN-B10 (left panel **a**), AN-E10 (middle panel **b**), and AN-IC10 (right panel **c**)), and extracted and calcined (AN-B10* (left panel **a**), AN-E10* (middle panel **b**), and AN-IC10* (right panel **c**)) samples. AN refers to aluminum nitrate.

**Figure 5:**
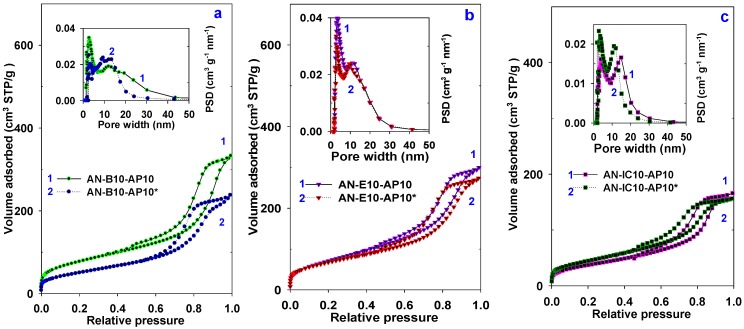
N_2_ adsorption isotherms and their corresponding PSD curves (insets) for extracted (AN-B10-AP10 (left panel **a**), AN-E10-AP10 (middle panel **b**), and AN-IC10-AP10 (right panel **c**)), and extracted calcined (AN-B10-AP10*(left panel **a**), AN-E10-AP10*(middle panel **b**), and AN-IC10-AP10*(right panel **c**)) samples. Note that AN and AP refer to aluminum nitrate and aminopropyl, respectively.

Figure 4 and Figure 5 show comparisons of N_2_ adsorption/desorption isotherms for the Al–MO samples synthesized using Al–NN as an alumina precursor with three different bridged organosilanes in the absence and presence of 3-aminopropyltriethoxysilane (AP) at −196 °C (77 K), respectively. All extracted (AN-B10, AN-E10, AN-IC10, AN-B10-AP10, AN-E10-AP10, AN-IC10-AP10), and extracted and calcined (AN-B10*, AN-E10*, AN-IC10*, AN-B10-AP10*, AN-E10-AP10*, AN-IC10-AP10*) samples exhibit type IV nitrogen adsorption isotherms with steep capillary condensation–evaporation steps beginning at a relative pressure of around 0.85 (see adsorption isotherms in Figure 4 and Figure 5). Insets in Figure 4 and Figure 5 show the pore size distribution (PSD) curves corresponding to the aforementioned adsorption isotherms obtained by the KJS method [53]. Our N_2_ adsorption isotherms reveal that the extracted samples derived from Al–NN (AN-IC10, AN-B10, AN-E10, AN-B10-AP10, AN-E10-AP10) have better surface properties, as evidenced by higher surface area, total pore volume (V_t_), and pore width (W_max_), as compared to the extracted and calcined counterparts (AN-IC10*, AN-B10*, AN-E10*, AN-B10-AP10*, AN-E10-AP10*). This may be attributed to the shrinkage of organosilica structure due to the calcination process at a relatively high temperature (350 °C) [54].

**Figure 6:**
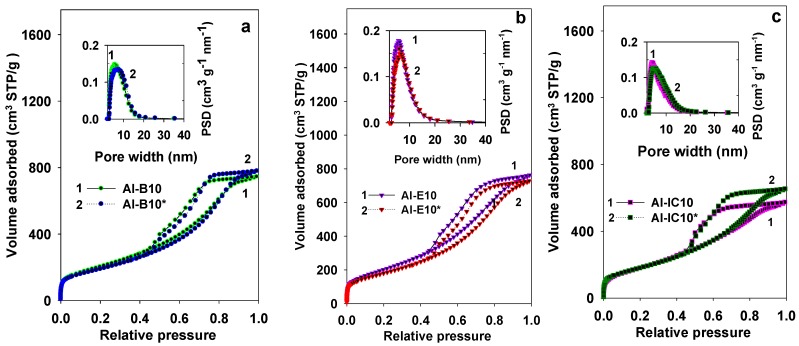
N_2_ adsorption isotherms and their respective PSD curves (insets) for the extracted (AI-B10 (left panel **a**), AI-E10 (middle panel **b**), and AI-IC10 (right panel **c**)), and extracted calcined (AI-B10* (left panel **a**), AI-E10* (middle panel **b**), AI-IC10* (right panel **c**)) samples, where B, E, and IC refer to benzene, ethylene, and isocyanurate bridging groups, respectively; AI refers to aluminum isopropoxide.

**Figure 7:**
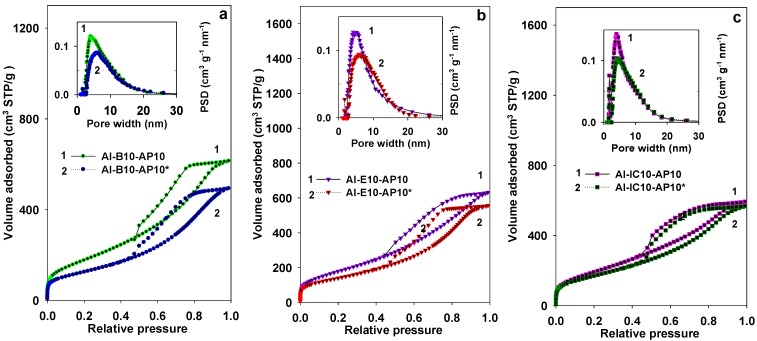
N_2_ adsorption isotherms (left panel) and the corresponding PSD curves (insets) for the extracted (AI-B10-AP10 (left panel **a**), AI-E10-AP10 (middle panel **b**), and AI-IC10-AP10 (right panel **c**)), and extracted calcined (AI-B10-AP10* (left panel **a**), AI-E10-AP10* (middle panel **b**), AI-IC10-AP10* (right panel **c**)) samples. AI refers to aluminum isopropoxide, and AP represents aminopropyl.

Similar to the Al–NN-derived samples, all Al–IP-derived samples also show type IV isotherm with relatively broad capillary condensation–evaporation steps pertained to H1 type hysteresis loop (see Figure 6 and Figure 7) and quite narrow PSDs (see insets in Figure 6 and Figure 7). As shown in Table 1, all extracted and calcined samples derived from Al–IP (AI-B10*, AI-E10*, AI-IC10*, AI-B10-AP10*, AI-E10-AP10*, and AI-IC10-AP10*) exhibit lower surface parameters, including surface area, microporosity, and pore width, as compared to the corresponding extracted samples (AI-B10, AI-E10, AI-IC10, AI-B10-AP10, AI-E10-AP10, and AI-IC10-AP10). For example, the specific surface area of AI-IC10 decreases from 655 m^2^·g^−1^ to 618 m^2^·g^−1^ after grafting aminopropyl hanging groups (AI-IC10-AP10*). In addition, the pore width of AI-IC10 sample reduces from 5.6 nm to 5.4 nm after introducing aminopropyl hanging groups (AI-IC10-AP10*), see Table 1. The reduction in the surface properties of the extracted and calcined samples is due to the shrinkage of the organosilica structure upon calcination at 350 °C [54].

It is noteworthy that the samples prepared from Al–IP precursor show much higher surface area and total pore volume (V_t_) as compared to the analogous samples prepared from Al–NN precursor, see Table 1 and Figure 7. For instance, the specific surface area of AI-B10 is 742 m^2^·g^−1^, as compared to 319 m^2^·g^−1^ of AN-B10, and the total pore volume (V_t_) of AI-B10 is 1.24 cm^3^·g^−1^ as compared to 0.63 cm^3^·g^−1^ of AN-B10, see Table 1. However, Al–NN-derived samples exhibit higher pore widths as compared to Al–IP-derived samples. For example, the pore width of AN-B10 sample is 13.6 nm as compared to 5.6 nm of AI-B10 sample.

**Figure 8:**
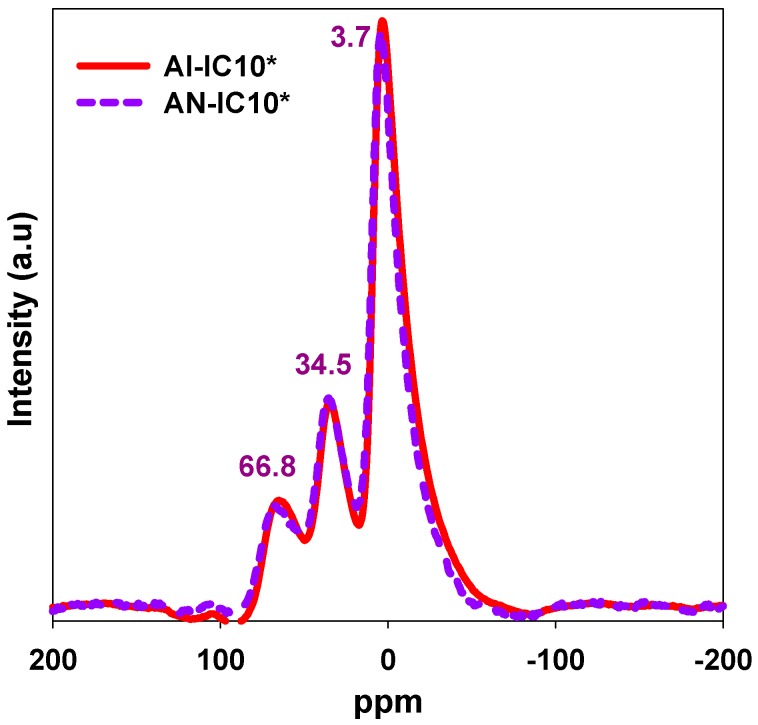
^27^Al-MAS NMR spectra of the AI-IC10* and AN-IC10* for extracted and calcined samples.

**Figure 9:**
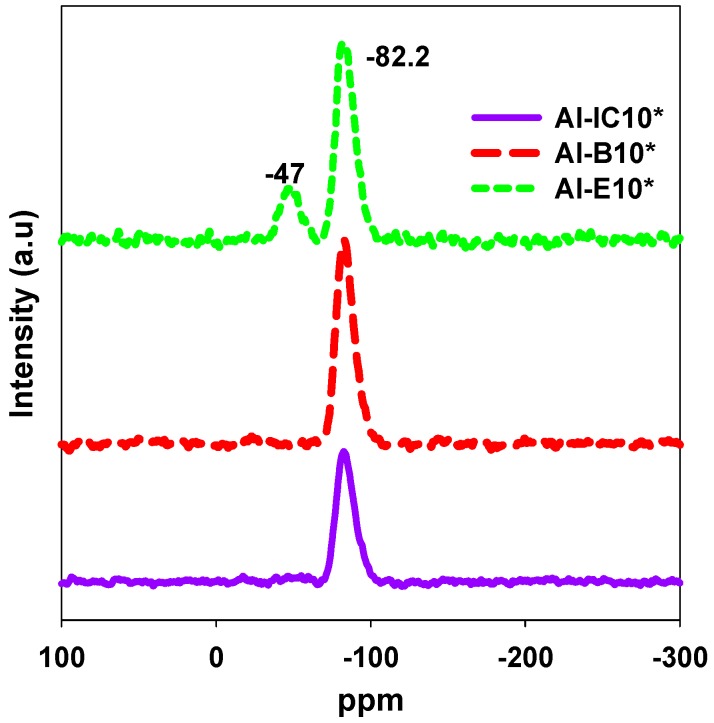
^1^H–^29^Si-MAS NMR spectra of the AI-IC10*, AI-B10*, and AI-E10* samples.

**Figure 10:**
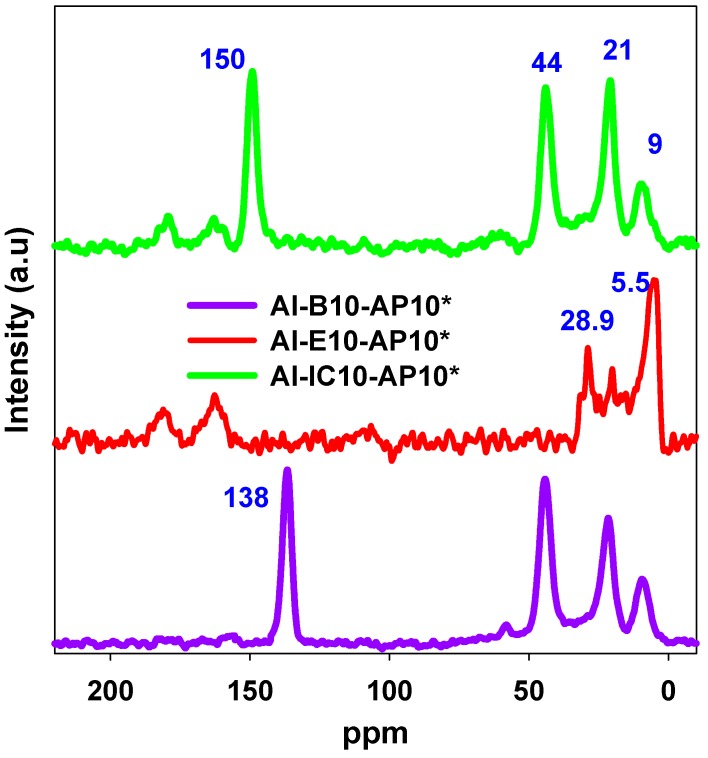
^1^H–^13^C CP/MAS NMR spectra of the AI-IC10-AP10*, AI-E10-AP10*, and AI-B10-AP10* samples.

^13^C, ^27^Al, and ^29^Si solid-state NMR spectra provide a comprehensive description of the chemical environment of carbon, aluminum, and silicon atoms present in the Al–MO materials studied. For instance, ^1^H–^29^Si cross polarization (CP) MAS NMR spectra reflect the degree of condensation of the siloxane bonds of isocyanurate, benzene, ethylene bridging groups, and aminopropyl hanging groups present in the hybrid organosilica framework. ^27^Al magic-angle spinning (MAS) NMR spectra reveal the coordination of the incorporated aluminum units in the Al–MO composites. ^1^H–^13^C CP/MAS NMR spectra confirm the presence of organic bridging (B, E, IC) and surface aminopropyl groups. The ^27^Al MAS NMR spectra exhibit three peaks for the Al–MO samples studied, see Figure 8. As shown in Figure 8, both AI-IC10* and AN-IC10* samples display an intense resonance peak at 3.7 ppm attributed to the aluminum species in an octahedral environment. The additional weak resonance peaks, visible at about 66.8 and 34.5 ppm, can be attributed to the aluminum in tetrahedral and pentahedral symmetries, respectively. The ^1^H–^29^Si CP/MAS NMR spectra of the AI-B10*, AI-E10*, and AI-IC10* samples show one prominent resonance peak at −82.2 ppm (Figure 9), corresponding to Q^2^ (Si–(OSi)_2_(OH)_2_)/(Si–(OSi)_2_(OH)(OAl)) groups. The spectrum of AI-E10* displays an additional small peak at ~−47 ppm, corresponding to the T^1^ (R–Si–(OSi)_1_(OH)_2_) structure. There are no visible peaks above 90 ppm, indicating the absence of Q^n^ (n = 3,4) structures. Figure 10 shows the ^1^H–^13^C CP/MAS spectra of the Al-IC10-AP10*, Al-B10-AP10*, and Al-E10-AP10* samples. The AI-IC10-AP10* and AI-B10-AP10* samples show three characteristic resonance peaks at 9, 21, and 44 ppm, referring to the carbon atoms bonded to Si atoms, carbon atoms present in the middle of propyl chain, and carbon atoms directly attached to N atoms, respectively. The characteristic sharp peaks at 150 and 138 ppm reflect carbon atoms in isocyanurate and benzene bridging groups, respectively. The spectrum of the AI-E10-AP10* sample exhibits four distinct peaks at 5.5, 9.0, 21.0, and 28.9 ppm. The first peak at 5.5 ppm refers to ethylene bridging groups. The second peak at 9 ppm refers to the carbon atoms present in the aminopropyl chain directly bonded to silicon. Note that the carbon atom directly bonded to Si also appears at 9 ppm. The third and fourth peaks at 21 and 28 ppm, of AI-E10-AP10* sample, correspond to the carbon atoms in the middle of the propyl chain and carbon atoms directly attached to N atoms in the aminopropyl chain, see Figure 10.

### 3.2. CO_2_ Sorption

**Figure 11:**
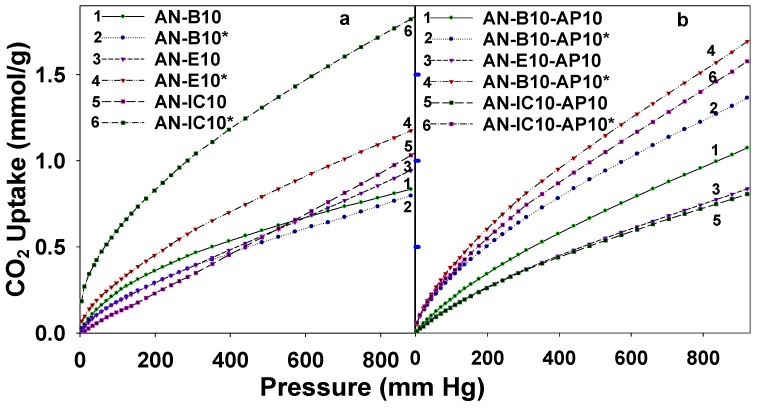
CO_2_ adsorption profiles at 25 °C, measured on the extracted, and extracted and calcined **a**) AN-Z10 and **b**) AN-Z10-AP10 samples, where Z = B, E, or IC.

**Figure 12:**
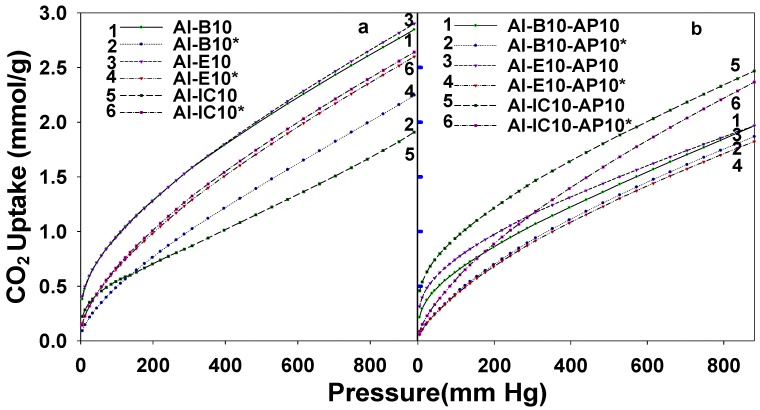
CO_2_ adsorption profiles at 25 °C measured on the extracted, and extracted and calcined (*) (**a**) AN-Z10 and (**b**) AN-Z10-AP10 samples, where Z = B, E, or IC.

Four series of Al–MO samples were examined for CO_2_ adsorption at pressures up to ~1.2 atm and 25 °C. Figure 11 and Figure 12 show a comparison of CO_2_ adsorption isotherms measured for all Al–MO samples studied. AN-B10, AN-E10, and AN-IC10 samples, and their extracted-followed-by-calcination counterparts (AN-B10*, AN-E10*, and AN-IC10*) show CO_2_ uptakes at 1 atm in the range from 0.72 to 1.72 mmol/g (Table 1 and Figure 11a). However, AI-B10, AI-E10, and AI-IC10 samples, and their extracted-followed-by-calcination counterparts (AI-B10*, AI-E10*, and AI-IC10*) exhibit higher CO_2_ uptakes at 1 atm, varying from 1.66 to 2.64 mmol/g (Table 1 and Figure 12a). 

It has been previously reported that the CO_2_ adsorption at ambient temperature is governed by a physisorption mechanism, which is dependent on the volume of micropores (below 2 nm), in particular, ultramicropores (below 0.7 nm) and surface area [55,56]. However, the samples studied show very low micropore volumes, suggesting that, in this case, the surface area and surface properties are major factors determining the CO_2_ adsorption (see Table 1). Moreover, the samples synthesized using Al–IP precursor show an approximately two-fold increase in the specific surface area and microporosity, in comparison to the corresponding samples synthesized from Al–NN precursor (see Table 1) and, consequently, their CO_2_ uptakes at 1 atm are higher. Our results also reveal that the structure of alumina precursor plays a vital role in determining the surface properties of the Al–MO hybrid materials. Therefore, aluminum isopropoxide (Al–IP) precursor works better than aluminum nitrate nanahydrate (Al–NN) precursor, due to its ability to form 3D extended mesoporous structures with block copolymers during the co-condensation process [54,57]. Among three silica precursors, organosilica composite with ethylene groups (AI-E10) exhibits the highest CO_2_ uptake of 2.64 mmol/g at 1 atm, see Table 1. At the same pressure, the samples with benzene (AI-B10) and isocyanurate (AI-IC10*) bridging groups exhibit the CO_2_ uptakes of 2.60 and 2.39 mmol/g, respectively. Our data suggest that the samples subjected to extraction and calcination have lower surface parameters, including microporosity, mesoporosity, surface area, and pore size (see Table 1), as compared to the samples subjected to extraction only, which is due to the shrinkage of the Al–MO materials at high temperatures. For example, the extracted and calcined samples with benzene and ethylene bridging groups show slightly lower CO_2_ adsorption capacities as compared to the samples subjected to extraction only. The CO_2_ uptakes measured at 1 atm for AI-B10 and AI-E10 change from 2.60 and 2.64 mmol/g, to 1.99 and 2.35 mmol/g, when calcination is performed (see Table 1). By contrast, the CO_2_ uptakes measured for the extracted and calcined samples with nitrogen-containing isocyanurate group (AN-IC10* and AI-IC10*) are larger than those for the extracted samples (AN-IC10 and AI-IC10), see Table 1. Higher CO_2_ adsorption on the AN-IC10* and AI-IC10* samples could be assigned to the unique structure of isocyanurate group and the presence of nitrogen species favoring interactions with CO_2_. Grafting aminopropyl hanging groups on the surface of the Al–MO composites did not cause a significant effect on the CO_2_ uptake (see Table 1). Al–MO samples with aminopropyl hanging groups also display lower microporosity and surface area (see Table 1 and Figure 11 and Figure 12). Our results demonstrate that the CO_2_ adsorption on mesoporous alumina– organosilica materials is mainly determined by the surface area of the sample, alumina precursor, and structure and functionality of the organic bridging groups. Calcination of the alumina–silica samples at 350 °C did not improve their adsorption properties. Amine-containing materials are commonly used as chemical sorbents at higher temperatures; however, grafting amine groups on the Al–MO materials did not affect physisorption of CO_2_ at the conditions studied. 

Cycle stability of the Al–MO material with the highest CO_2_ adsorption capacity was also tested as described previously [58,59]. Cycle stability measurements for AI-E10 were conducted for six consecutive cycles, and CO_2_ uptake was measured. The CO_2_ uptake remained stable after six adsorption/desorption cycles with approximately 2.64 mmol/g CO_2_ uptake at 1 atm, suggesting good stability of AI-E10 materials. Note that the alumina samples (extracted and calcined at 350 °C) do not show a long-range order on the XRD profiles; thus, they are amorphous in nature (data not shown). 

Alumina-based composite materials have been investigated for CO_2_ capture. Table 2 summarizes a comparison of CO_2_ uptakes for various alumina-based composite materials reported in the literature at low and elevated temperatures. However, studies of alumina-based materials for CO_2_ capture at ambient conditions are rare. The sorbents studied in this work show much higher CO_2_ uptake at 25°C, as compared to the values reported previously for alumina-based materials under the same conditions.

## 4. Conclusions

Mesoporous alumina–organosilica (Al–MO) materials exhibited high CO_2_ adsorption capacity at 25 °C. CO_2_ adsorption properties of Al–MO materials depend on the surface area of the sample, alumina precursor, and structure and functionality of the organosilica bridging group. The Al–NN- and Al–IP-derived samples exhibited the highest CO_2_ uptakes of 1.72 and 2.64 mmol/g at 1 atm, respectively. High CO_2_ uptake, reusability, selectivity, and good thermal and mechanical stability, make Al–MO materials potential adsorbents for larger-scale CO_2_ capture at 25 °C.

## Figures and Tables

**Table 1:** Adsorption parameters obtained for the alumina–silica samples studied.

Samples	S_BET_ (m^2^/g)	V_sp_ (cc/g)	V_t_ (cc/g)	V_mi_ (cc/g)	W_max_ (nm)	*n*_CO2_ (mmol/g)	n*_CO2_ (µmol/m^2^)
AN-B10	319	0.60	0.63	0.02	2.9/13.6	0.78	2.45
AN-B10*	150	0.42	0.44	<0.01	12.0^#^	0.73	4.87
AN-E10	393	0.91	0.94	<0.01	4.1/17.0	1.09	2.77
AN-E10*	219	0.67	0.72	<0.01	15.9^#^	0.85	3.88
AN-IC10	289	0.74	0.77	0.01	4.0/16.4^#^	0.92	3.18
AN-IC10*	286	0.68	0.68	<0.01	4.0/13.2^#^	1.72	6.01
AN-B10-AP10	284	0.50	0.52	0.02	2.9/12.3	0.96	3.38
AN-B10-AP10*	186	0.36	0.38	<0.01	9.4/13.3	1.22	6.56
AN-E10-AP10	265	0.46	0.48	<0.01	3.4/11.6^#^	0.74	2.79
AN-E10-AP10*	247	0.42	0.43	<0.01	3.2/9.8^#^	1.51	6.11
AN-IC10-AP10	138	0.26	0.26	<0.01	4.1/14.8	0.72	5.21
AN-IC10-AP10*	163	0.24	0.24	<0.01	3.6/11.1	1.40	8.59
AI-B10	742	1.14	1.24	0.05	5.1	2.60	3.50
AI-B10*	684	1.20	1.28	0.03	6.1	1.99	2.91
AI-E10	740	1.18	1.27	0.03	5.7	2.64	3.57
AI-E10*	652	1.12	1.19	0.02	6.1	2.35	3.60
AI-IC10	664	0.89	0.95	0.04	4.1	1.66	2.50
Al-IC10*	655	1.01	1.06	0.02	4.7	2.39	3.65
AI-B10-AP10	654	0.94	1.00	0.02	4.2	1.83	2.80
AI-B10-AP10*	452	0.76	0.78	<0.01	5.6	1.74	3.85
AI-E10-AP10	664	0.97	1.03	0.02	4.0	1.86	2.80
AI-E10-AP10*	509	0.86	0.90	<0.01	5.1	1.70	3.34
AI-IC10-AP10	713	0.91	0.98	0.03	4.0	2.33	3.27
AI-IC10-AP10*	618	0.87	0.90	0.02	5.4	2.21	3.58

S_BET_—specific surface area, V_sp_—single point pore volume, V_t_—total pore volume, V_mi_—volume of micropores, W_max_—pore width (^#^ refers to the middle value of the “flat” PSD peak), *n*_CO2_—number of moles of CO_2_ adsorbed at 1 atm per gram of the sample, and n*_CO2_—number of moles of CO_2_ adsorbed at 1 atm per unit surface area of the sample. B, E, or IC refer to benzene, ethylene, and isocyanurate bridging groups, respectively; AN and AP refer to aluminum nitrate and aminopropyl, respectively. Extracted, and extracted and calcined samples, are denoted without* and with*, respectively.

**Table 2:** Comparison of the CO_2_ sorption data for different alumina-based composite materials.

Material	Temperature (°C)	Pressure (atm)	CO_2_ Uptake (mmol/g)	Ref.
Al–Mg oxide	60 (13%H_2_O)	0.99	1.36	[49]
Al_2_O_3_–silica	120	0.99	2.20	[50]
Al–Zr–mixed oxide–silica	0 25 60 120	1 1 1 1	1.83 1.39 2.60 2.37	[58]
Al–Mg mixed oxide–silica	300	0.99	0.46	[60]
γ-Al_2_O_3_	60	9.9	1.94	[61]
N-doped γ-Al_2_O_3_	55	NG	0.67	[62]
Al–Mg mixed oxide–nitrate–graphene oxide	60	1	1.00	[63]
Al_2_O_3_–silica	25	1	2.64	This work
